# [Retracted] MicroRNA‑124 acts as a tumor‑suppressive miRNA by inhibiting the expression of Snail2 in osteosarcoma

**DOI:** 10.3892/ol.2023.14041

**Published:** 2023-09-04

**Authors:** Jianghong Huang, Yujie Liang, Meiquan Xu, Jianyi Xiong, Daping Wang, Qiang Ding

Oncol Lett 15: 4979–4987, 2018; DOI: 10.3892/ol.2018.7994

Following the publication of this paper, it was drawn to the Editor's attention by a concerned reader that certain of the tumor images shown in Fig. 6B on p. 4985 were strikingly similar to tumor images that appeared, albeit in different form, in another article written by different authors at different research institutes [Zhang L, Lu Y, Yue X, Li H, Luo X, Wang Y, Wang K and Wan J: MiR-124 suppresses growth of human colorectal cancer by inhibiting STAT3. PLoS One 8: e70300, 2013].

Owing to the fact that the contentious data in the above article had already been published elsewhere prior to its submission to *Oncology Letters*, the Editor has decided that this paper should be retracted from the Journal. The authors were asked for an explanation to account for these concerns, but the Editorial Office did not receive a reply. The Editor apologizes to the readership for any inconvenience caused.

